# Vitamin K3 (Menadione) Targets TBK1 to Enhance the Efficacy of Pirfenidone in a Mouse Model of Bleomycin-Induced Pulmonary Fibrosis

**DOI:** 10.3390/ijms27219739

**Published:** 2026-10-30

**Authors:** Yanan Wang, Junxian Cao, Yingxin Zhou, Xuxia Cao, Shiwen Deng, Yu Li, Caifeng Li, Shihuan Tang, Peng Chen

**Affiliations:** 1 State Key Laboratory for Quality Ensurance and Sustainable Use of Dao-Di Herbs, Institute of Chinese Materia Medica, China Academy of Chinese Medical Sciences, Beijing 100700, China; 2 Experimental Research Center, China Academy of Chinese Medical Sciences, Beijing 100700, China; 3 Jiangxi Province Key Laboratory of Traditional Chinese Medicine Pharmacology, Institute of Traditional Chinese Medicine Health Industry, China Academy of Chinese Medical Sciences, Nanchang 330115, China

**Keywords:** idiopathic pulmonary fibrosis, vitamin K3, naphthoquinone, pirfenidone, bleomycin, mouse model, pulmonary fibrosis, TBK1, STAT3 signaling

## Abstract

Idiopathic pulmonary fibrosis (IPF) is a progressive and irreversible interstitial lung disease. Current antifibrotic drugs, exemplified by pirfenidone (PFD), only slow disease progression with limited efficacy and adverse effects, underscoring the need for novel therapeutic strategies. By integrating single-cell transcriptomics with the Drug2Cell algorithm, we performed drug prioritization at single-cell resolution and identified menadione (vitamin K3, VK3), a synthetic naphthoquinone and the minimal core of naturally occurring vitamin K, as a candidate preferentially active in alveolar epithelial cells. In bleomycin (BLM)-stimulated A549 cells and a mouse model of pulmonary fibrosis, therapeutic co-administration of low-dose VK3 and PFD reduced vimentin expression by 44.8% and collagen deposition by 50.3%, and decreased fibroblast abundance by 36.7%, thereby attenuating histopathological damage and restoring BLM-disrupted alveolar epithelial homeostasis. Experiments with VK3 alone further demonstrated that its antifibrotic activity is independent of conversion to vitamin K2. Mechanistically, thermal proteome profiling identified TANK-binding kinase 1 (TBK1) as the principal candidate target; molecular docking and molecular dynamics simulations predicted a stable binding mode, independently confirmed by biolayer interferometry and differential scanning fluorimetry, demonstrating direct binding of VK3 to TBK1 with nanomolar affinity. *TBK1* knockdown suppressed BLM-induced fibrotic responses, establishing TBK1 as a positive regulator of pulmonary fibrosis. In vitro signaling analysis revealed that VK3 plus PFD inhibits phosphorylation of TBK1 and STAT3, whereas PFD alone reduces phosphorylation of JAK1 and STAT3, suggesting that both agents converge on STAT3 through distinct upstream nodes. Although VK3 exhibits dose-dependent cytotoxicity precluding direct clinical use as monotherapy, its validated high-affinity target TBK1 and privileged naphthoquinone scaffold lay a rational foundation for lead optimization toward safer anti-IPF agents, and its ability to enhance PFD efficacy supports further development as an adjunct to existing antifibrotic therapy.

## 1. Introduction

Idiopathic pulmonary fibrosis (IPF) is a progressive and irreversible interstitial lung disease characterized by excessive extracellular matrix (ECM) deposition and destruction of alveolar architecture, with a median survival of only 2.5 to 3.5 years after diagnosis [1]. Currently approved antifibrotic agents, including pirfenidone (PFD), nintedanib, and the recently FDA-approved nerandomilast, slow disease progression but fail to reverse established fibrosis or substantially improve survival [2,3]. PFD, the most widely prescribed first-line therapy, exerts broad antifibrotic and anti-inflammatory effects through suppression of TGF-β1 and CTGF signaling, fibroblast proliferation, and collagen deposition [4,5,6,7]. However, its clinical benefit remains modest, and dose-limiting adverse effects including gastrointestinal intolerance, photosensitivity, and fatigue frequently compromise patient compliance [8,9]. Moreover, PFD monotherapy cannot fully disrupt the multifactorial inflammatory and fibrotic network underlying IPF, underscoring an urgent need for novel therapeutic strategies, including agents that can enhance the efficacy of existing antifibrotics.

In response to the limitations of single-target interventions, hybrid bifunctional molecules have emerged as a promising strategy to simultaneously modulate multiple interconnected fibrotic pathways. Among these, multi-target epigenetic–kinase hybrid compounds have attracted considerable attention, especially dual inhibitors designed to target histone deacetylases (HDACs) and pro-fibrotic tyrosine kinases such as EGFR. Such hybrid molecules integrate two pharmacophores into a single scaffold, enabling concurrent suppression of epigenetic remodeling and EGFR-dependent profibrotic signaling in experimental models of pulmonary fibrosis (PF) [10]. While these hybrid molecules demonstrate attractive multi-pathway inhibitory potential, their development is often hindered by complicated organic synthesis, poor cell-type selectivity, and off-target toxicity. Alternative strategies are therefore required to discover bioactive small molecules capable of synergizing with approved antifibrotic drugs to interrupt fibrotic cascades.

Transcriptome-guided drug repositioning offers an efficient and cost-effective route to identify candidate agents for complex diseases. Drug2Cell, a computational framework that integrates single-cell transcriptomic disease signatures with drug-induced transcriptional profiles, enables cell-type-resolved prediction of therapeutic efficacy across large compound libraries [11,12]. Applying this approach to public single-cell RNA-seq datasets from the lungs of patients with IPF [13], we screened 2381 compounds and identified vitamin K3 (VK3, menadione), a synthetic naphthoquinone and the minimal core of naturally occurring vitamin K, as a candidate with predicted activity preferentially in alveolar epithelial cells, a critical cell type driving IPF pathogenesis.

Emerging clinical evidence links vitamin K metabolism to pulmonary fibrosis. Warfarin, a vitamin K antagonist, is associated with increased IPF mortality, and genetic variants in vitamin K cycle genes such as *VKORC1* and *CYP2C9* correlate with familial IPF susceptibility [14,15,16]. VK3 is a synthetic naphthoquinone best known as the in vivo precursor to biologically active vitamin K2 (menaquinones), naturally occurring congeners synthesized by gut microbiota and enriched in fermented foods [17,18]. Beyond this well-characterized metabolic role, VK3 possesses a naphthoquinone scaffold that is widely represented among bioactive natural products [19]. Consistent with this chemotype, VK3 exhibits documented anti-inflammatory and antioxidant activity both in vitro and in vivo [20,21] and has shown protective effects in models of acute lung injury and renal interstitial fibrosis, where it engages disease-specific targets distinct from those of conventional antifibrotics [22,23,24]. However, whether VK3 is efficacious in IPF and through which direct molecular target it acts remain unknown. TANK-binding kinase 1 (TBK1), a central regulator of innate immune and inflammatory signaling, has been implicated in the pathogenesis of fibrosis [25,26,27]. Nevertheless, small-molecule compounds that directly target TBK1 and their therapeutic potential for IPF remain largely unexplored.

Bleomycin (BLM), an antineoplastic glycopeptide antibiotic originally isolated from *Streptomyces verticillus*, is a standard component of chemotherapeutic regimens for Hodgkin lymphoma and testicular cancer. Its antitumor activity is mediated by oxidative DNA damage resulting from reactive oxygen species generated by a BLM–iron–oxygen complex. Because the lung expresses low levels of bleomycin hydrolase, the principal enzyme that inactivates BLM, the drug accumulates preferentially in pulmonary tissue and induces epithelial and endothelial injury, interstitial pneumonitis, and progressive fibrosis [28]. This dose-limiting pulmonary toxicity has been exploited to establish the most widely used experimental model of PF in rodents [29]. Although BLM-induced PF does not fully recapitulate the chronic, progressive course and usual interstitial pneumonia (UIP) histology of human IPF, it reproduces key pathogenic features of the disease, including alveolar epithelial injury, fibroblast activation, and excessive ECM deposition, and thus remains the standard preclinical model for evaluating candidate antifibrotic therapies [30]. Accordingly, the antifibrotic efficacy of VK3 was evaluated in the BLM-induced PF mouse model. Here, we demonstrate that VK3 directly binds TBK1 with nanomolar affinity and enhances the antifibrotic efficacy of PFD in vitro and in vivo through suppression of TBK1 and downstream STAT3 signaling, validating TBK1 as a therapeutic target in IPF and providing a target-validated foundation for naphthoquinone-based lead optimization.

## 2. Results

### 2.1. Single-Cell Transcriptomic Profiling and Drug2Cell-Based Screening of VK3

**Figure 1:**
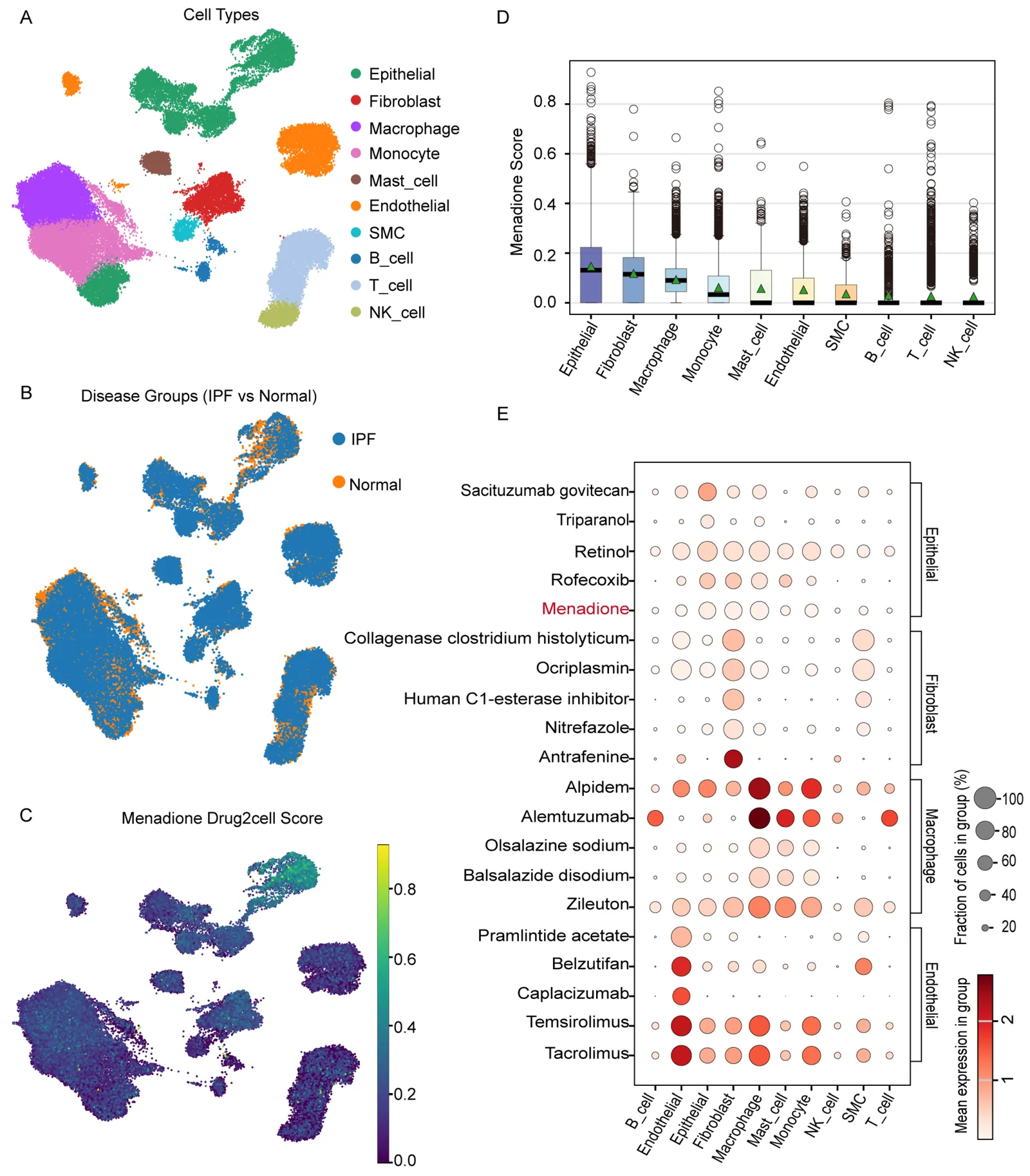
Screening of VK3 by integrating public single-cell sequencing data with the Drug2cell method. (**A**) UMAP visualization of all major cell types, showing 10 distinct cell populations. (**B**) UMAP plot colored by disease status (Normal vs. IPF). (**C**) UMAP plot of VK3 sensitivity scores. (**D**) Box plot showing the distribution of Menadione sensitivity scores across different cell types. Each open circle corresponds to the sensitivity score of an individual cell; the black line inside the box denotes the median, and the green triangle indicates the mean. Colored boxes are used to distinguish distinct cell subsets. (**E**) Bubble plot of predicted drug sensitivity scores for four major fibrosis-associated cell clusters (epithelial cells, fibroblasts, macrophages, and endothelial cells).

After stringent quality control, a total of 55,015 high-quality cells were obtained and manually annotated into ten major cell types: epithelial cells, fibroblasts, macrophages, monocytes, mast cells, endothelial cells, smooth muscle cells, B cells, T cells, and NK cells (Figure 1A). The UMAP plot colored by disease status revealed the distribution of cells from normal lung tissue and IPF samples, providing disease context for subsequent drug sensitivity analysis (Figure 1B and Figure S1). Using the Drug2cell algorithm, activity scores for 2381 drugs were calculated by quantifying the per-cell enrichment of drug target gene sets.

Among all evaluated compounds, VK3 exhibited a markedly cell-type-specific sensitivity profile. Epithelial cells showed the highest mean sensitivity score (0.147 ± 0.135), followed by fibroblasts (0.118 ± 0.098), both of which fell into the high-sensitivity group (score > 0.10). Macrophages and monocytes displayed moderate sensitivity, whereas immune cells and vascular cells consistently showed low sensitivity (Figure 1D). Notably, Figure 1E presents drug sensitivity scores for four core cell types closely associated with pulmonary fibrosis (epithelial cells, fibroblasts, macrophages, and endothelial cells). The predicted sensitivity of VK3 in epithelial cells ranks among the highest across all 2381 screened compounds (Figure 1E). The complete sensitivity dataset covering all ten cell clusters is available in Supplementary Figure S2. The UMAP plot of VK3 sensitivity scores further illustrated this cell-type-specific distribution pattern (Figure 1C). It is well established that epithelial cells and fibroblasts are key drivers of the initiation and progression of pulmonary fibrosis. Based on these single-cell predictive results and a literature review, we selected VK3 as a candidate compound for subsequent in vitro and in vivo experimental validation to evaluate its potential therapeutic value in fibrotic diseases.

### 2.2. Low-Dose VK3 Enhances the Antifibrotic Efficacy of PFD In Vitro

**Figure 2:**
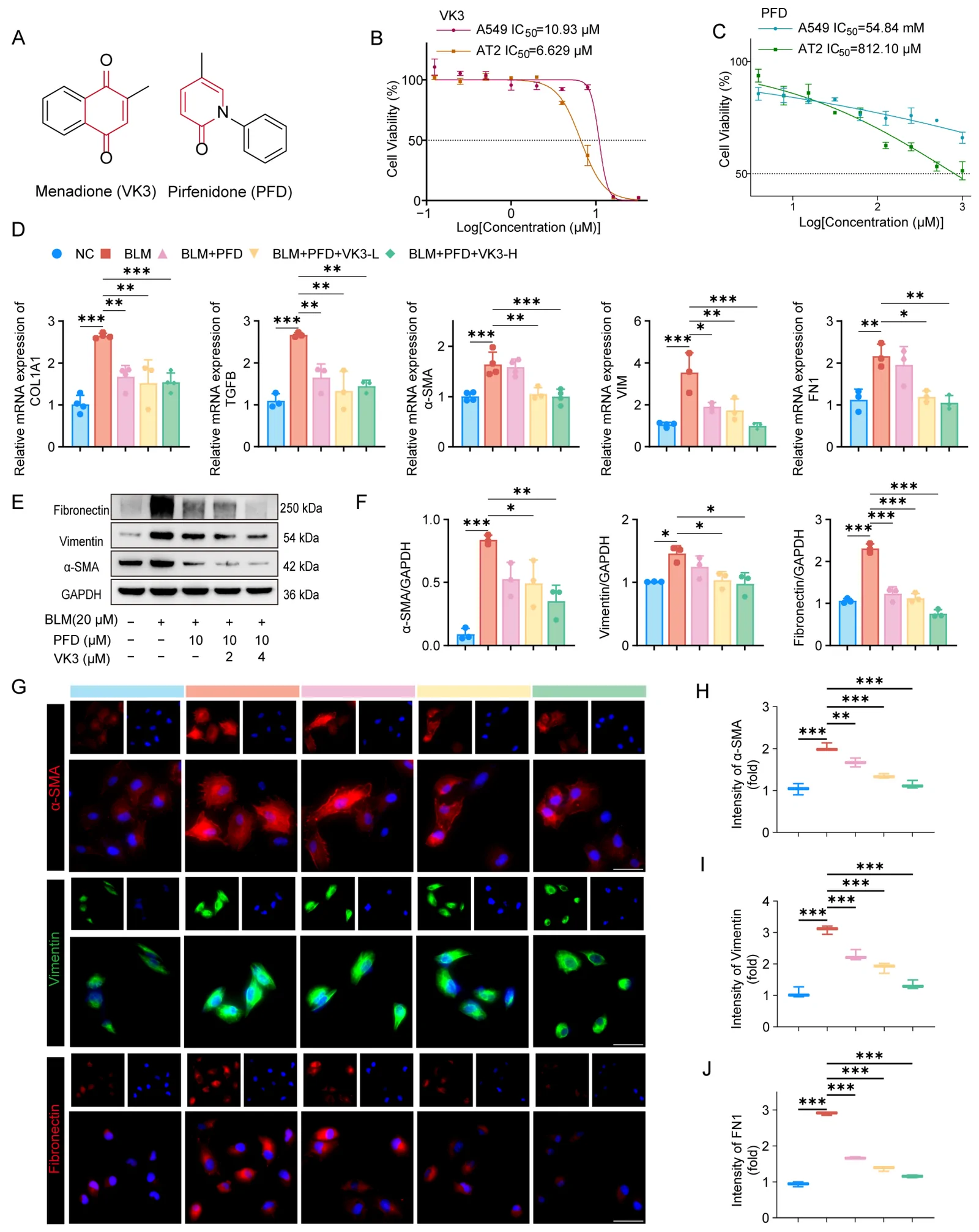
Low-dose VK3 potentiates pirfenidone-mediated suppression of BLM-induced fibrotic responses in vitro. (**A**) Chemical structures of VK3 and PFD (red rings highlight the shared pharmacophore: quinone/pyridone core). (**B**) IC_50_ values of VK3 in A549 and AT2 cells. (**C**) IC_50_ values of PFD in A549 and AT2 cells. (**D**) Relative mRNA expression of COL1A1, FN1, VIM, α-SMA, and TGF-β in A549 cells treated with BLM or in combination with PFD and/or VK3 (Blue, NC; red, BLM; pink, BLM+PFD; yellow, BLM+PFD+VK3-L; green, BLM+PFD+VK3-H). (**E**) Representative WB showing protein levels of α-SMA, VIM, and FN in A549 cells under indicated treatments. (**F**) Quantification of protein bands normalized to GAPDH. (**G**) Immunofluorescence staining of α-SMA, VIM, and FN in A549 cells. Scale bar = 50 μm. (**H**–**J**) Quantification of fluorescence intensity for α-SMA (**H**), Vimentin (**I**), and FN1 (**J**). * *p* < 0.05, ** *p* < 0.01, *** *p* < 0.001 vs. BLM group.

To explore the potential of VK3 synergizing with PFD for IPF treatment, an in vitro fibrosis model was established using A549 cells, a well-characterized alveolar epithelial cell line. Cells were exposed to bleomycin (BLM, 20 μM) for 24 h to induce fibrotic responses, followed by treatment with PFD alone (200 μM) or PFD combined with low-dose (1 μM) or high-dose (2 μM) VK3 for an additional 24 h. During fibrogenesis, α-smooth muscle actin (α-SMA) and vimentin (VIM) serve as key markers of myofibroblast activation and epithelial–mesenchymal transition (EMT). Collagen type I (*COL1A1*) and fibronectin (*FN1*) are major components of excessive extracellular matrix (ECM) deposition. Transforming growth factor-β1 (TGF-β1) is a master regulator that drives the expression of these fibrotic proteins and promotes ECM synthesis and fibrosis progression [31]. As shown in Figure 2A,B, BLM stimulation significantly increased the protein levels of COL1A1, α-SMA, VIM, and FN compared with the control group. TGF-β1 protein levels showed a similar upward trend. Treatment with PFD alone at a low dose effectively inhibited these BLM-induced changes, reducing the expression of all fibrotic markers. Notably, the combination of low-dose VK3 (1 μM) with PFD produced a marked effect, further decreasing the levels of COL1A1, α-SMA, VIM, FN, and TGF-β1 compared with PFD monotherapy. The high-dose VK3 combination group showed even greater suppression of the fibrotic markers; notably, the low-dose combination alone was already sufficient to significantly reduce BLM-induced expression of all fibrotic markers (COL1A1, α-SMA, VIM, FN, and TGF-β1) compared with PFD monotherapy.

### 2.3. Low-Dose VK3 and PFD Enhance Efficacy Against BLM-Induced PF in Mice

**Figure 3:**
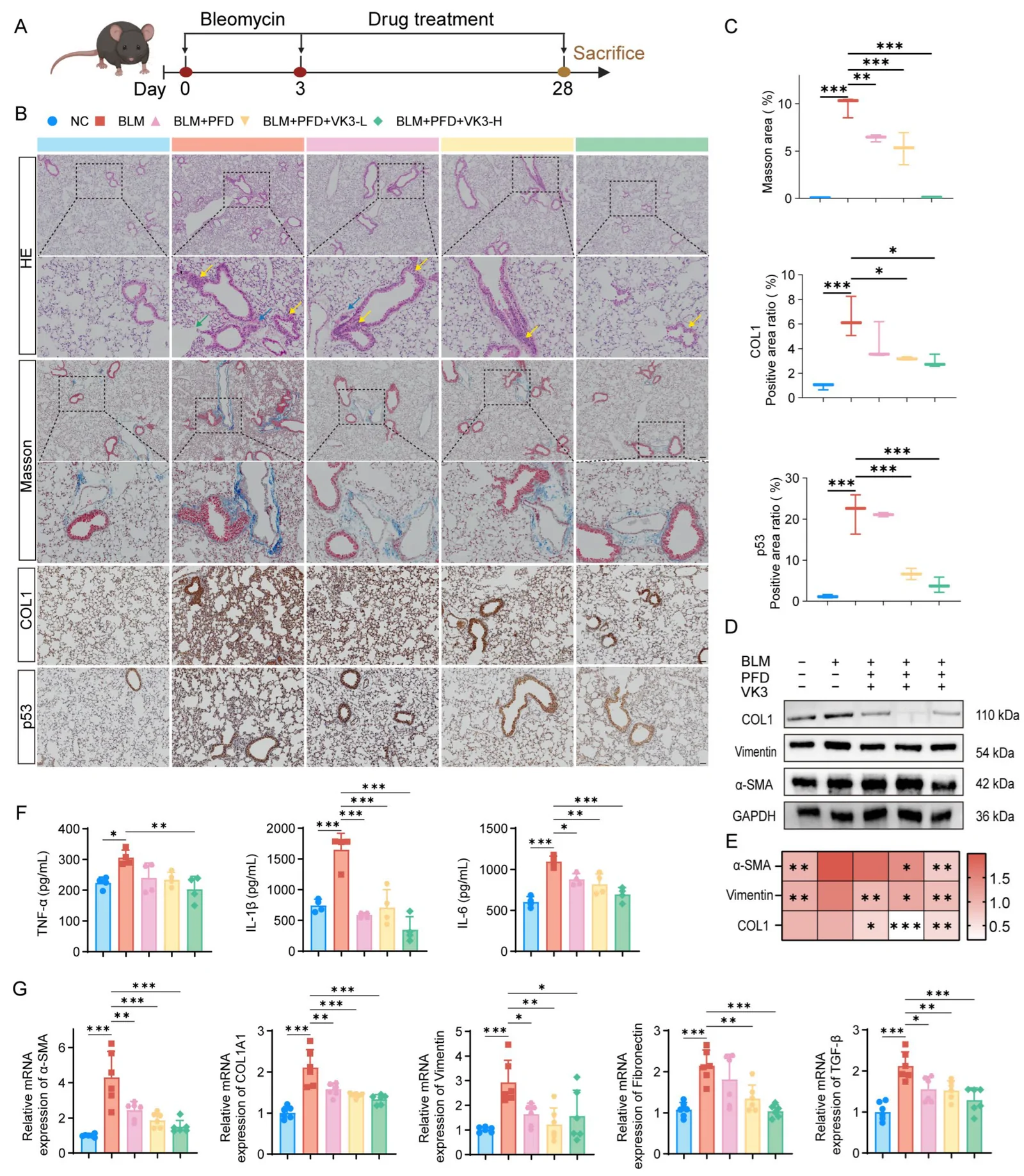
Low-dose VK3 plus PFD mitigates BLM-induced PF in mice. (**A**) Schematic diagram of the animal experimental protocol. (**B**) Representative images of H&E, Masson’s trichrome, COL1A1, and p53 immunohistochemical (IHC) staining of lung tissue sections. In H&E images: yellow arrows indicate inflammatory cell infiltration; blue arrows indicate thickened alveolar septa; green arrows indicate alveolar fusion and alveolar wall rupture. In Masson’s trichrome staining, blue signals represent collagen deposition. Scale bar = 50 μm. (**C**) Quantitative analysis of Masson’s trichrome-positive area, COL1A1, and p53 staining intensity (Blue, NC; red, BLM; pink, BLM+PFD; yellow, BLM+PFD+VK3-L; green, BLM+PFD+VK3-H.). (**D**) WB analysis of COL1A1, VIM, and α-SMA protein expression in mouse lung tissues. (**E**) Quantification of protein bands normalized to GAPDH. (**F**) Serum levels of TNF-α, IL-6, and IL-1β measured by ELISA. (**G**) Relative mRNA expression of α-SMA, COL1A1, VIM, FN, and TGF-β in lung tissues determined by RT-qPCR. * *p* < 0.05, ** *p* < 0.01, *** *p* < 0.001 vs. BLM group.

The BLM-induced pulmonary fibrosis model was selected as the in vivo model for this study because it reliably reproduces key pathological features of human IPF and is widely recognized [32,33]. Figure 3A illustrates the establishment of the BLM-induced pulmonary fibrosis mouse model and the experimental treatment schedule. Representative H&E and Masson’s trichrome staining were performed to evaluate lung pathological injury and fibrotic remodeling. Compared with the control group, BLM challenge induced severe destruction of lung architecture. In magnified H&E images, red arrows indicate robust inflammatory cell infiltration, blue arrows show thickened alveolar septa, and green arrows highlight alveolar fusion and alveolar wall rupture. Masson staining revealed extensive blue-stained collagen deposition in BLM-injured lung tissues. PFD intervention markedly ameliorated these pathological manifestations, attenuating lung tissue injury, inflammatory infiltration, and collagen accumulation. Immunohistochemical results demonstrated that the protein expression levels of COL1A1 and p53 in fibrotic lung tissues were markedly increased relative to the normal control group, while PFD treatment reduced their expression. Notably, low-dose VK3 combined with PFD produced enhanced therapeutic effects (Figure 3B,C). In the BLM-induced pulmonary fibrosis model, the early acute inflammatory response is considered a key trigger for subsequent fibrosis [34]. Serum levels of TNF-α, IL-6, and IL-1β were significantly elevated in the BLM group. PFD treatment significantly reduced these cytokine levels, and the PFD plus high-dose VK3 group showed even greater inhibition (Figure 3F). This suggests that combination therapy enhances the inhibitory effect of PFD on inflammatory cytokines. Western blot and RT-qPCR results showed that the combination also produced similar trends for COL1A1, fibrosis markers (α-SMA, VIM, FN), and the key profibrotic cytokine TGF-β. The combination group exhibited better antifibrotic efficacy than PFD alone (Figure 3D,E,G). In summary, low-dose VK3 combined with PFD enhances therapeutic efficacy against BLM-induced PF in mice.

### 2.4. VK3 and PFD Reshape the Single-Cell Transcriptomic Landscape in Mouse PF

**Figure 4:**
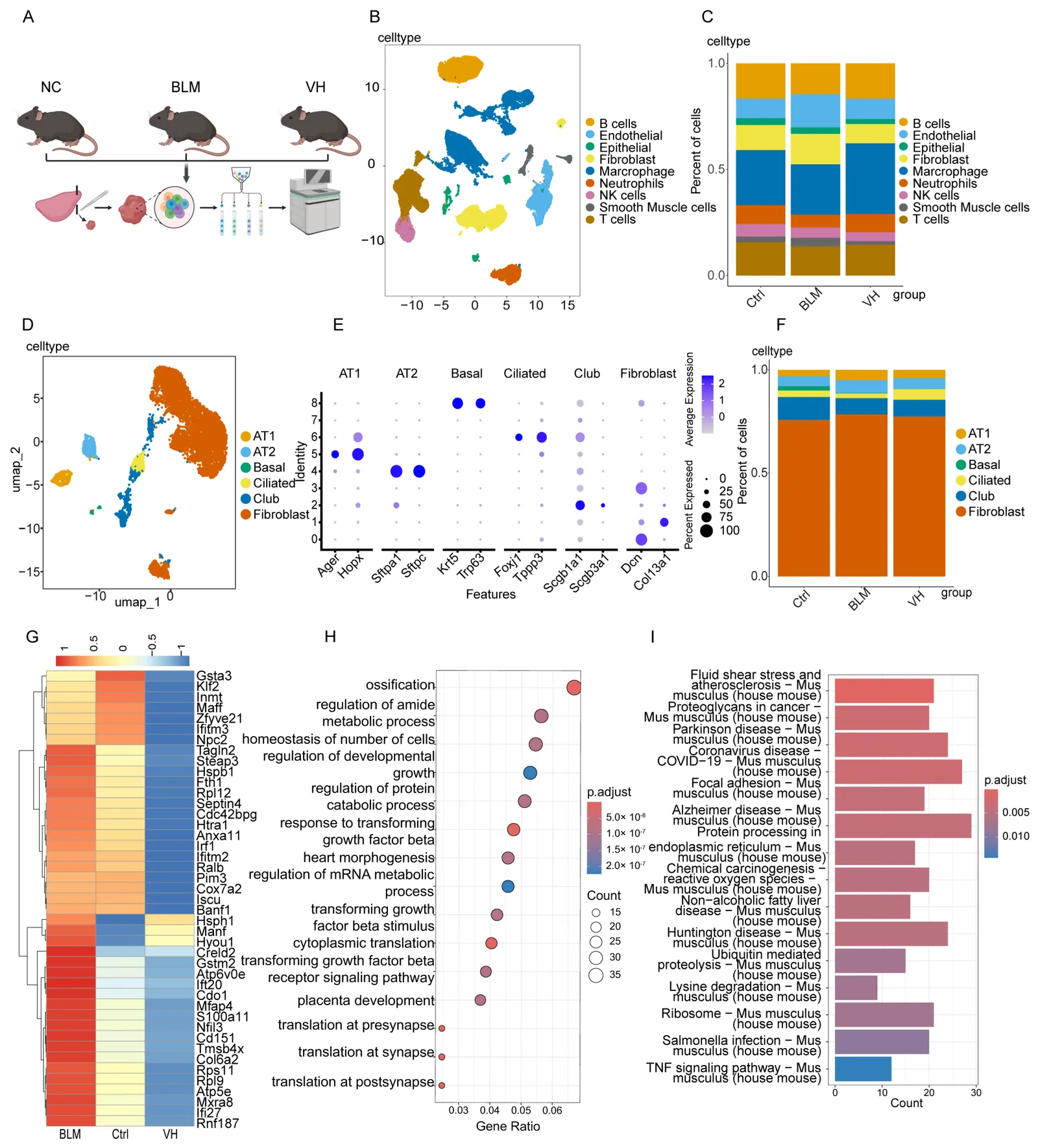
VK3 and PFD remodel the scRNA-seq landscape in fibrotic mouse lungs. (**A**) Schematic illustration of the experimental design for scRNA-seq (created with BioRender). (**B**) UMAP plot of all major cell types. (**C**) Relative abundance of major cellular subtypes. (**D**) UMAP plot of epithelial cell subtypes. (**E**) Dot plot showing the expression levels of representative marker genes for each epithelial subtype. (**F**) Proportional abundance of epithelial cell subtypes. (**G**) Heatmap of DEGs associated with pulmonary fibrosis across groups. (**H**) GO enrichment analysis of hub genes. (**I**) KEGG pathway enrichment analysis of hub genes.

To elucidate the in vivo mechanism of VK3, single-cell RNA sequencing (scRNA-seq) was performed on lung tissues from control, BLM model, and VK3 high-dose combination groups (Figure 4A). After strict quality control and data preprocessing, comprehensive cell type annotation was conducted. This revealed the effects of VK3 on cell composition and transcriptional landscape in the fibrotic lung microenvironment (Figure S1B). UMAP dimensionality reduction identified nine major cell populations (Figure 4B): B cells, endothelial cells, epithelial cells, fibroblasts, macrophages, neutrophils, smooth muscle cells, NK cells, and T cells. Analysis of cell type proportion changes among groups showed that, compared with the control group, the BLM group had significantly increased proportions of fibroblasts and smooth muscle cells, decreased epithelial cells, and increased macrophage infiltration. These changes are consistent with tissue injury, stromal activation, and fibrosis formation. The VK3 high-dose combination group significantly reversed these changes, reducing fibroblasts and smooth muscle cells, increasing epithelial cells, and restoring cell composition toward the control group, indicating an antifibrotic effect (Figure 4C).

Further subtyping of epithelial cells identified the following subsets: AT1 (*AGER*, *HOPX*), AT2 (*SFTPA1*, *SFTPC*), basal cells (*KRT5*, *TRP63*), ciliated cells (*FOXJ1*, *TPPP3*), club cells (*SCGB1A1*, *SCGB3A1*), and fibroblasts (*DCN*, *COL13A1*) (Figure 4D,E). Cell abundance comparison showed that, compared with the control group, the BLM group had increased proportions of AT1 and AT2 cells; decreased club, ciliated, and basal cells; and a marked increase in fibroblasts. This indicates epithelial subtype imbalance and excessive stromal activation, typical features of fibrosis. In the VK3 high-dose group, fibroblast proportions decreased and epithelial subtype distribution normalized, suggesting that VK3 treatment ameliorates fibrosis-related epithelial–mesenchymal dysregulation (Figure 4F). The heatmap in Figure 4G shows that the VK3 high-dose group significantly reduced the elevated expression of fibrosis-related genes (*COL6A2*, *TMSB4X*, *Mfap4*, *ANXA11*, *HTRA1*, *TAGLN2*) induced by BLM.

GO enrichment analysis showed that differentially expressed genes in the model group were most significantly enriched in the TGF-β response and its receptor signaling pathway. This pathway is widely recognized as a major regulator of pulmonary fibrosis, participating in myofibroblast differentiation and ECM deposition. Other biological processes involved include regulation of proteolysis, cell number homeostasis, developmental growth, and ossification. These findings indicate abnormal activation of the TGF-β signaling axis, mediating fibroblast activation, excessive ECM deposition, and aberrant tissue remodeling, which are fully consistent with the core pathological processes of fibrosis (Figure 4H). KEGG enrichment analysis revealed that differentially expressed genes were mainly enriched in pathways such as focal adhesion, TNF signaling, protein processing in endoplasmic reticulum, ribosome, and ubiquitin-mediated proteolysis. These pathways regulate cell adhesion, chronic inflammation, matrix protein synthesis, and metabolic dysregulation, further driving the development and progression of fibrosis (Figure 4I). Overall, the enrichment results are highly consistent with the molecular mechanisms of organ fibrosis.

### 2.5. Identification of TBK1 as the Primary Direct Target of VK3

**Figure 5:**
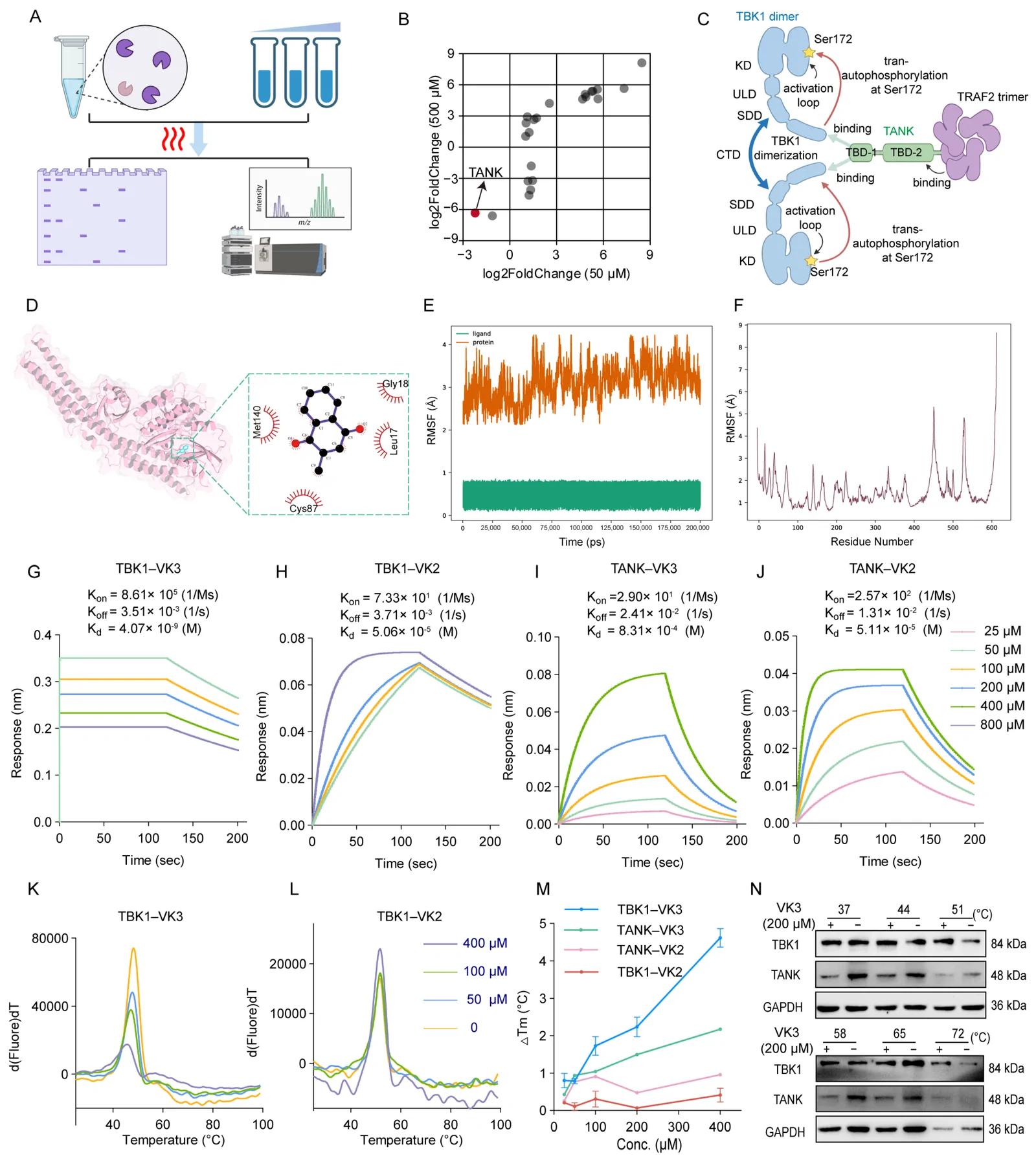
VK3 directly targets TBK1 with high affinity. (**A**) Schematic workflow of TPP for identifying VK3-binding proteins (created with BioRender). (**B**) TPP results identifying TANK as a potential VK3-interacting protein. (**C**) Schematic diagram of the TBK1–TANK–TRAF2 ternary complex. The scaffold protein TANK recruits TRAF2 and forms a ternary complex with TBK1, which triggers TBK1 oligomerization and autophosphorylation at Ser172. TBK1 dimer: light blue; TANK: light green; TRAF2 trimer: light purple. Yellow stars mark the Ser172 phosphorylation sites. KD, kinase domain; ULD, ubiquitin-like domain; SDD, scaffold dimerization domain; CTD, C-terminal domain; TBD, TBK1-binding domain. (**D**) 2D representation of molecular docking showing the interaction between VK3 and the TBK1 kinase domain. (**E**,**F**) RMSD plots from molecular dynamics simulations. (**G**–**J**) BLI binding curves of TBK1–VK3 (**G**), TBK1–VK2 (**H**), TANK–VK3 (**I**) and TANK–VK2 (**J**). (**K**,**L**) DSF thermal stability curves of TBK1–VK3 (**K**), TBK1–VK2 (**L**). (**M**) Quantification of Δ*T*m for TBK1 and TANK induced by VK3 and VK2. (**N**) Temperature-dependent CETSA showing the thermal stabilization of TBK1 and TANK in A549 cell lysates treated with VK3.

To further elucidate the molecular target underlying the antifibrotic effect of VK3, TPP was performed and identified TRAF family member-associated NF-κB activator (TANK) as a potential binding protein of VK3 (Figure 5A,B). TANK acts as a scaffold protein: its N-terminal domain binds to the C-terminal dimerization domain of TBK1, while its C-terminal domain recruits TRAF2, thereby forming a TBK1–TANK–TRAF2 ternary complex. This complex drives TBK1 oligomerization and trans-autophosphorylation at Ser172, which is essential for TBK1 activation [35]. Given that TANK directly regulates TBK1 kinase activity, we hypothesized that TBK1 serves as a key downstream mediator of the antifibrotic effect of VK3 (Figure 5C). To verify direct binding between VK3 and TBK1, molecular docking was first conducted. A docking score below −6.0 kcal·mol^−1^ is commonly considered an empirical threshold for potential binding activity [36]. The docking score of VK3 with the kinase pocket of TBK1 was −8.2 kcal·mol^−1^, indicating strong potential binding (Figure 5D). Molecular dynamics simulation confirmed stable complex formation, with key interactions involving MET140 (Figure 5E,F).

Given that VK3 is rapidly converted to VK2 (MK-4) by UBIAD1 in vivo [37], we included VK2 in our in vitro binding assays to determine whether the direct antifibrotic effect is mediated by VK3 itself or its metabolite (Figure S4H). The binding of both compounds to recombinant TBK1 and TANK proteins was evaluated using DSF and BLI. BLI measurements revealed that VK3 binds to TBK1 with high affinity, exhibiting a *K*_d_ of 4.07 nM, a rapid association rate, and a slow dissociation rate (Figure 5G). In contrast, VK2 showed much weaker binding to TBK1, with a *K*_d_ of 50.6 μM (Figure 5H). For TANK, the *K*d values for VK3 and VK2 were 831 μM and 51.1 μM, respectively (Figure 5I,J). DSF analysis further supported these findings: 400 μM VK3 decreased the melting temperature (*T*m) of TBK1 by 4.4 °C and that of TANK by 2.1 °C, indicating a more pronounced thermal destabilization of TBK1 upon VK3 binding (Figure 5K–M). Similar DSF results for TANK with VK3 and VK2 are presented in Figure S1. Temperature-dependent cellular thermal shift assays (CETSAs) further corroborated these observations (Figure 5N). WB results (Figure S2) showed that VK3 and VK3 + simvastatin both significantly suppressed VIM and α-SMA expression with comparable efficacy, whereas VK2 alone exhibited little effect. Since simvastatin blocks the conversion of VK3 to VK2 by inhibiting *UBIAD1* [17], these results exclude the possibility that VK3 must be converted to VK2 to exert its antifibrotic effect. Collectively, these findings indicate that the antifibrotic effect of VK3 primarily depends on its parent form directly targeting TBK1, rather than through metabolism to VK2 or engagement of TANK.

### 2.6. TBK1 Knockdown Inhibits BLM-Induced Fibrotic Responses

**Figure 6:**
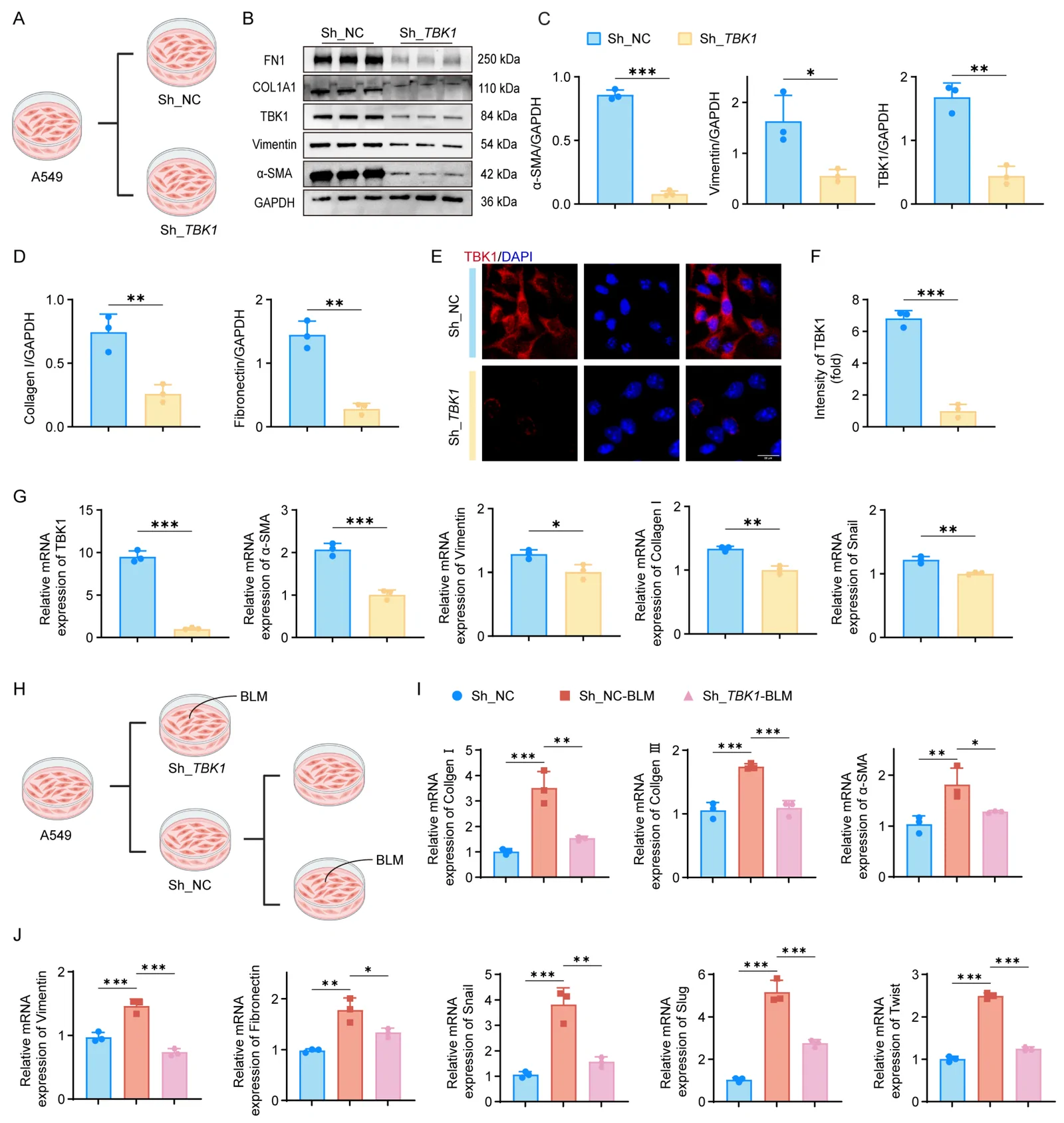
Knockdown of TBK1 abrogates BLM-induced fibrotic and EMT phenotypes in A549 cells. (**A**) Schematic illustration of the experimental design: cells were transduced with sh_NC or sh_*TBK1* lentivirus (created with BioRender). (**B**) Representative Western blots showing the protein levels of TBK1, α-SMA, VIM, COL1, and FN in cells stably expressing sh_NC or sh_*TBK1*. (**C**) Quantification of protein expression of α-SMA, VIM, and TBK1 normalized to GAPDH. (**D**) Quantification of COL1 and FN protein levels normalized to GAPDH. (**E**) IF staining of TBK1 in sh_NC- and sh_TBK1-expressing cells. Scale bar = 20 μm. (**F**) Quantification of TBK1 fluorescence intensity from (**E**). (**G**) RT-qPCR analysis of mRNA levels of *TBK1*, α-SMA, VIM, *COL1A1*, and Snail. (**H**) Experimental scheme: cells stably infected with sh_NC or sh_TBK1 were treated with BLM for 48 h (created with BioRender). (**I**) RT-qPCR analysis of *COL1A1*, *COL3A1*, and α-SMA mRNA levels. (**J**) RT-qPCR analysis of VIM, FN, Snail, Slug, and *Twist* mRNA levels. * *p* < 0.05, ** *p* < 0.01, *** *p* < 0.001.

We designed and constructed a recombinant lentiviral vector expressing short hairpin RNA targeting TBK1 (sh*TBK1*). A lentivirus carrying a scrambled sequence (shNC) served as the negative control (Figure 6A). Cells were transduced with these vectors, respectively. As shown in Figure 6B,G, both protein and mRNA levels of TBK1 were significantly reduced in sh*TBK1*-treated cells compared with shNC-treated cells, confirming stable knockdown efficiency. Consistently, the protein levels of α-SMA, VIM, COL1, and FN were also markedly decreased upon TBK1 knockdown (Figure 6B–D). Immunofluorescence staining further confirmed the reduction in TBK1 expression in sh*TBK1*-expressing cells (Figure 6E,F). Moreover, RT-qPCR analysis revealed that knockdown of TBK1 significantly suppressed the mRNA levels of *TBK1*, α-SMA, VIM, *COL1A1*, and *Snail* (Figure 6G).

### 2.7. TBK1 Knockdown Alleviates BLM-Induced Fibrotic Responses and Synergizes with PFD via the JAK–STAT Pathway

**Figure 7:**
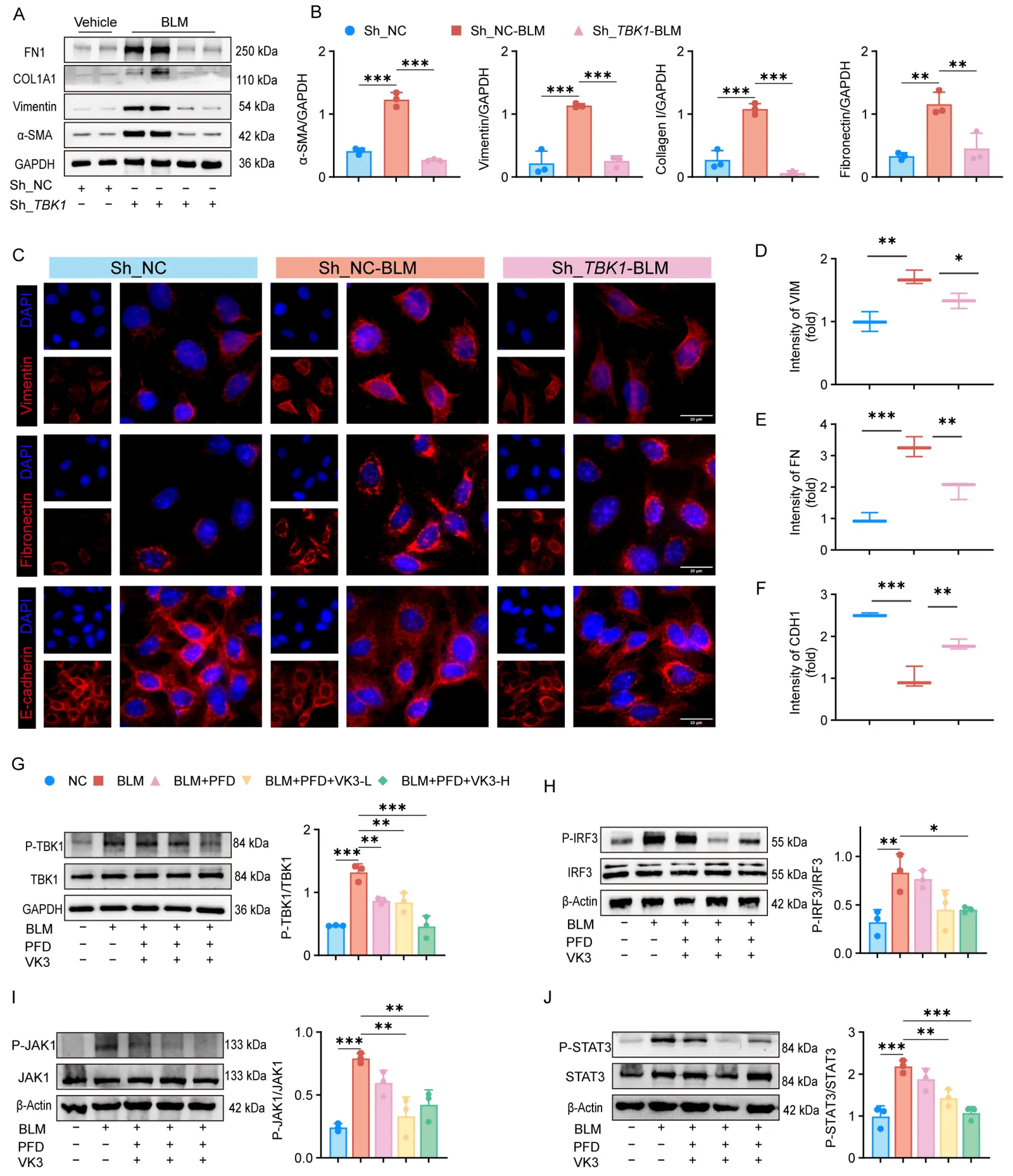
VK3 potentiates the antifibrotic effect of PFD via the TBK1–JAK–STAT signaling axis. (**A**) Representative WB showing the protein levels of α-SMA, VIM, COL1, and FN in cells stably infected with sh_NC or sh_*TBK1* lentivirus, followed by treatment with BLM. (**B**) Quantification of the protein expression levels from A, normalized to GAPDH (Blue, Sh_NC; orange, Sh_NC-BLM; pink, Sh_TBK1-BLM). (**C**) IF staining of VIM, FN and E-Cadherin in the indicated groups. Scale bar = 20 μm. (**D**–**F**) Quantification of fluorescence intensity for VIM, FN, and E-Cadherin from (**C**). (**G**–**J**) WB analysis of lung tissue homogenates from the animal experiment. Densitometric quantification of phosphorylated and total protein levels is presented as the ratio of phosphorylated to total protein: p-TBK1/TBK1 (**G**), p-IRF3/IRF3 (**H**), p-JAK1/JAK1 (**I**), p-STAT3/STAT3 (**J**). * *p* < 0.05, ** *p* < 0.01, *** *p* < 0.001.

To further evaluate the effect of TBK1 knockdown on the BLM-induced fibrotic response, cells stably transduced with shNC or sh*TBK1* lentivirus were treated with BLM for 48 h according to the experimental scheme (Figure 6H). BLM stimulation markedly upregulated the mRNA levels of *COL1A1*, *COL3A1*, and α-SMA in the shNC group compared with untreated cells, whereas this upregulation was significantly attenuated in the sh*TBK1* group (Figure 6I). Similarly, the BLM-induced increases in VIM, FN, Snail, Slug, and Twist were also abrogated by TBK1 knockdown (Figure 6J). Consistently, VK3 combined with PFD suppressed fibrotic protein expression (α-SMA, VIM, COL1, FN) and restored E-cadherin in a TBK1-dependent manner (Figure 7A–F).

We next examined the phosphorylation status of TBK1, JAK1, IRF3, and STAT3 in lung tissues from the BLM-induced mouse model (Figure 7G–J). Compared with the NC group, the BLM model group exhibited significantly elevated levels of p-TBK1, p-JAK1, p-IRF3, and p-STAT3. PFD monotherapy significantly reduced p-TBK1 and p-STAT3 levels. VK3 combination treatment (especially at the high dose) further decreased p-TBK1, p-JAK1, and p-STAT3 levels on top of PFD, with the inhibitory effects on p-TBK1 and p-STAT3 showing dose-dependent enhancement. Moreover, the high-dose VK3 combination group also significantly reduced p-IRF3 levels.

## 3. Discussion

The present study identifies VK3 as a potential candidate agent that enhances the antifibrotic efficacy of PFD through single-cell transcriptome-guided drug repositioning and establishes TBK1 as its direct high-affinity target. Pirfenidone monotherapy exhibits limited therapeutic potency for IPF, and accumulating preclinical evidence demonstrates that combining pirfenidone with additional bioactive agents can produce enhanced antifibrotic effects, representing a promising strategy to improve therapeutic outcomes [38,39,40]. The novelty of this work lies in the integrated strategy combining single-cell transcriptome mining for drug repurposing and multi-orthogonal target validation, which identifies VK3, a natural naphthoquinone compound, as a nanomolar-affinity direct binder of TBK1. Reported synthetic TBK1 inhibitors have mostly been evaluated as monotherapy in preclinical pulmonary fibrosis models, and no combination studies with pirfenidone for pulmonary fibrosis have been described to date. In contrast, our study demonstrates that VK3 can potentiate pirfenidone-mediated antifibrotic effects. To our knowledge, this is the first report revealing that VK3 augments PFD activity via the TBK1–STAT3 axis in pulmonary fibrosis, providing a new combinatory therapeutic concept for IPF. The core finding that VK3 directly binds TBK1, supported by orthogonal approaches including thermal proteome profiling, molecular docking, molecular dynamics simulations, biolayer interferometry, and differential scanning fluorimetry, provides a mechanistic explanation for the PFD-enhancing activity of VK3 and identifies a tractable target for subsequent medicinal chemistry optimization. Functionally, TBK1 knockdown phenocopied key antifibrotic effects of VK3, confirming TBK1 as a positive regulator of pulmonary fibrosis. Using VK3 alone in mechanistic experiments, we further demonstrated that the antifibrotic effect of VK3 does not require its conversion to vitamin K2, confirming that VK3 itself, rather than its downstream metabolite, is the active species that engages TBK1. In addition, low-dose VK3 in combination with PFD exerted enhanced antifibrotic efficacy both in vitro and in vivo, and single-cell transcriptomic analysis revealed that the combination remodeled the cellular ecosystem of the fibrotic lung by reducing fibroblast abundance and restoring alveolar epithelial homeostasis.

The identification of TBK1 as a direct target of VK3 is consistent with the emerging role of TBK1 in fibrotic disorders. While TBK1 is classically known as a central kinase in innate immune signaling downstream of the STING and MAVS pathways, recent studies have implicated TBK1 in the pathogenesis of pulmonary fibrosis, systemic sclerosis, and osteoarthritis. Beyond canonical STING-dependent type-I interferon responses, TBK1 also modulates cytokine-dependent cell-death thresholds and cross-talks with JAK–STAT signaling cascades in disease contexts [41]. While TBK1 inhibitors have demonstrated antifibrotic activity in preclinical lung fibrosis models [25,38], a small-molecule candidate discovered through systematic target identification and validated as a direct TBK1 binder for IPF therapy remains lacking. Our study fills this gap by demonstrating that VK3, a naphthoquinone with documented anti-inflammatory activity, directly binds TBK1 with nanomolar affinity and enhances the antifibrotic efficacy of PFD in vitro and in vivo. The relationship between TBK1 and STAT3 appears context-dependent and phosphorylation-site-specific. A previous study reported that, under cytosolic DNA stimulation, TBK1 phosphorylates STAT3 at Ser754, which promotes STAT3 nuclear export and thereby inhibits its transcriptional activity [42]. In contrast, our data show that TBK1 knockdown suppresses fibrotic responses and that VK3-mediated TBK1 inhibition reduces STAT3 phosphorylation at Tyr705, the activating phosphorylation event that drives STAT3 nuclear translocation and fibrotic gene transcription. These findings indicate that, in the context of pulmonary fibrosis, TBK1 functions as a positive regulator of STAT3-driven fibrotic signaling. This discrepancy may reflect differences in STAT3 phosphorylation site, upstream stimulus, or cellular context, and warrants further investigation.

An important additional finding of this study is that low-dose VK3 enhances the antifibrotic efficacy of PFD both in vitro and in vivo. The JAK–STAT signaling pathway serves as a key convergent point downstream of multiple pro-fibrotic stimuli [43,44,45], and PFD has been reported to modulate this pathway in multiple disease models. In silicosis and bleomycin-induced pulmonary fibrosis models, PFD reduced lung fibrosis by inhibiting JAK2/STAT3 signaling [46,47], and in a chronic pancreatitis study, PFD reduced pro-inflammatory cytokine expression and inhibited M1 macrophage polarization via the JAK1/STAT3 pathway [48]. In our model, PFD reduced phosphorylation of both JAK1 and STAT3, although the suppression of p-JAK1 was incomplete. This observation may reflect indirect modulation of JAK1 signaling by PFD, suboptimal dosing, or context-dependent pathway activation, and the underlying mechanism requires further investigation. The cross-regulatory relationship between TBK1 and the JAK–STAT pathway provides a mechanistic rationale for the observed enhancement of antifibrotic efficacy by combination treatment. TBK1 has been linked to JAK/STAT activation in osteoarthritis and systemic sclerosis [49,50], and our data show that VK3 suppresses both p-TBK1 and p-STAT3. We therefore propose that VK3 and PFD converge on STAT3 through distinct upstream nodes, with VK3 acting primarily through TBK1 and PFD acting primarily through JAK1, and that simultaneous engagement of both nodes results in more complete suppression of STAT3-driven fibrotic transcription than either agent alone.

Based on our data and the existing literature, we propose a working model for the complementary actions of VK3 and PFD. In the fibrotic lung microenvironment, TBK1 and JAK1 represent two distinct upstream kinases that converge on STAT3 activation, with potential cross-regulatory interactions between them. The experimentally supported core of this model, namely that VK3 inhibits TBK1 and PFD modulates JAK1 with both converging on STAT3 suppression, has been detailed above. Beyond these experimentally supported observations, we further speculate that TBK1 inhibition by VK3 may restore the physiological function of IRF3, which has been reported to intersect with TBK1 signaling in fibrotic conditions [51], and that restored IRF3 activity may induce IFN-β and subsequent *SOCS3* expression, forming a feedback loop that indirectly suppresses JAK1 signaling [52]. In support of this possibility, we observed that higher-dose VK3 in combination with PFD reduced p-IRF3 levels, which is consistent with altered IRF3 signaling upon combined treatment and warrants further mechanistic investigation. However, this proposed IRF3–IFN-β–SOCS3 axis remains speculative and will require direct experimental validation, including measurement of IFN-β and SOCS3 expression, IRF3 nuclear translocation assays, and loss-of-function studies using IFN-β neutralizing antibodies or *SOCS3* knockdown.

Several limitations of this study should be acknowledged. First, mechanistic uncertainties remain: it remains unclear whether TBK1 directly phosphorylates STAT3 at Tyr705 or acts via intermediate kinases, and whether PFD modulates TBK1 and JAK1 through direct binding or indirect pathways; the incomplete suppression of p-JAK1 by PFD cannot be fully explained by current data. Our efficacy experiments also lacked a dedicated VK3 monotherapy group, so the standalone antifibrotic potency of VK3 cannot be quantified. In addition, the pharmacokinetic profile and chronic toxicity of VK3 have not been fully characterized, and its dose-dependent cytotoxicity represents a barrier for clinical translation. The in vitro experiments were performed using A549 cells, a human alveolar type II (ATII)-like cell line that is widely used to model fibrotic responses of the alveolar epithelium [53,54,55]. Although A549 cells retain certain ATII features, including multilamellar inclusion bodies [55], we acknowledge that a transformed cell line cannot fully recapitulate the biology of primary ATII cells, which are of limited availability and rapidly lose their differentiated phenotype under conventional culture conditions [56,57,58]. Validation in primary human ATII cells would therefore provide additional human-relevant evidence, and we have prioritized this in our ongoing follow-up studies. The present study also did not include validation in human pluripotent stem cell (hPSC)-derived alveolar models. iPSC-derived alveolar platforms, such as the induced respiratory airway progenitor (iRAP) system recently reported by Pezet et al. [59], represent valuable and promising tools for human-relevant IPF drug discovery and validation, and incorporating such systems into our future work is an important direction for further strengthening the translational significance of our findings.

Several limitations inherent in the experimental systems for assessing VK3 antifibrotic activity must also be acknowledged. Pulmonary fibrosis is a complex, multicomponent disease, and no single experimental system can fully recapitulate its pathological features [32]. Although in vitro monolayer cultures are valuable for elucidating the molecular mechanisms underlying the action of VK3, they provide a simplified representation of the tissue architecture of the fibrotic lung, lacking the three-dimensional alveolar–capillary structure, the biomechanical properties of the remodeled extracellular matrix, and the epithelial–mesenchymal–immune interactions that collectively drive fibrogenesis [60]. Furthermore, cell-based assays are unable to reflect a systemic organismal response, including systemic inflammation, immune cell recruitment, and pharmacokinetic–pharmacodynamic relationships. To mitigate these limitations, the in vitro mechanistic studies were complemented with an established in vivo model of pulmonary fibrosis, which retains organ-level tissue architecture and permits partial assessment of systemic immune responses. Nevertheless, animal models are likewise subject to inherent constraints in recapitulating the full spectrum of human IPF [61]. Importantly, these limitations do not undermine the central finding of this study, which is that VK3 exerts its antifibrotic activity through direct binding to TBK1, as evidenced by the in vitro binding assays. Future mechanistic and translational directions include: (1) establishing VK3 monotherapy groups to quantify its standalone antifibrotic activity and define optimal combination dosing with PFD; (2) validating findings in primary human ATII cells and hPSC-derived alveolar organoids to improve translational relevance; (3) resolving whether TBK1 directly or indirectly modulates STAT3 Tyr705 phosphorylation; (4) characterizing VK3 pharmacokinetics and long-term toxicity; (5) performing structure-guided modification of the VK3 naphthoquinone scaffold to generate novel TBK1 binders with lower cytotoxicity and higher binding affinity.

## 4. Materials and Methods

### 4.1. Cell Lines and Reagents

The A549 cell line (RRID: CVCL_0023) was obtained from the Shanghai Jinyuan Biotechnology Co., Ltd. (Beijing, China). Cells were cultured in RPMI 1640 medium supplemented with 1% penicillin-streptomycin at 37 °C in a humidified atmosphere containing 5% CO_2_. Vitamin K3 (VK3, purity > 98%) was purchased from Beijing InnoChem Science & Technology Co., Ltd. (Beijing, China). Bleomycin sulfate (BLM, 1.5–2.0 units/mg) and pirfenidone (PFD, purity > 98%) were purchased from Shanghai Macklin Biochemical Co., Ltd. (Shanghai, China).

### 4.2. Animal Procedures

Male C57BL/6 mice, aged 6–8 weeks and weighing 20 ± 2 g, were purchased from SPF Biotechnology Co., Ltd. (Beijing, China). Mice were kept at a constant temperature of 22 ± 2 °C and relative humidity of 50 ± 10%, under a 12 h light/dark cycle with food and water available ad libitum. Six mice were housed per cage, and bedding was replaced every 3 days. General health including activity, food intake and breathing was visually inspected daily, and body weight was recorded every 3 days throughout the experiment. The cages were checked daily for aggressive behavior; animals with fighting-related skin lesions were separated immediately. All experimental procedures conformed to the guidelines of the Ethics Committee of the China Academy of Chinese Medical Sciences (ERCCACMS11-2504-05), and every effort was made to reduce animal suffering. After 3 days of acclimatization, all mice were weighed, numbered in ascending order of body weight, and randomly allocated to five groups using a random number table, with six mice per group: (1) control group, (2) BLM group, (3) BLM + PFD group, (4) BLM + PFD + low-dose VK3 (VK3L) group, and (5) BLM + PFD + high-dose VK3 (VK3H) group. To establish a mouse model of idiopathic pulmonary fibrosis, mice in BLM groups were exposed to aerosolized BLM (4.5 mg/kg) once daily on days 1–3 [61,62]. Mice in the control group received aerosolized normal saline under the same schedule. PFD and VK3 were dissolved in normal saline containing 2% DMSO and 2% Tween-80 with ultrasonication to facilitate solubilization. BLM was prepared in sterile normal saline. The drug doses and administration schedules of PFD, VK3 and BLM were adopted from previously published studies. From day 4 to day 28, mice in treatment groups received once-daily intraperitoneal injection of corresponding regimens: PFD alone (50 mg/kg), PFD combined with VK3L (VK3 at 5 mg/kg), or PFD combined with VK3H (VK3 at 10 mg/kg) [22,63]. Mice in control and BLM groups received intraperitoneal injection of an equal volume of the above drug vehicle. All mice were euthanized at the end of day 28.

### 4.3. Lung Histological Analysis

After orbital blood collection, mice were euthanized, and the lungs were perfused with normal saline via the right ventricle. One portion of the lung tissue was fixed in 4% paraformaldehyde for at least 24 h, dehydrated, embedded in paraffin, and cut into 2–4 μm serial sections for H&E, Masson, and IHC staining. Three sections per specimen were examined, and six randomly selected fields per section were imaged under a light microscope. For IHC, sections underwent heat-induced antigen retrieval in EDTA buffer (pH 9.0), blocking with 3% H_2_O_2_ and 3% BSA, incubation with the primary antibody overnight at 4 °C and an HRP-conjugated secondary antibody, and DAB development with hematoxylin counterstaining. Masson staining was performed using a Masson’s Trichrome Stain Kit (Solarbio, Beijing, China; Cat# G1340). The percentages of collagen-positive area and DAB-positive area were quantified using ImageJ (v1.8.0). The remaining lung tissue was stored at −80 °C until PCR and Western blot analysis.

### 4.4. ELISA Analyses

Serum concentrations of tumor necrosis factor-alpha (TNF-α), interleukin-1β (IL-1β), and interleukin-6 (IL-6) were measured using commercial sandwich enzyme-linked immunosorbent assay (ELISA) kits (BioQuanti, Wuhan, Hubei, China; TNF-α: Cat# BQE-M0187-CM; IL-1β: Cat# BQE-M0105; IL-6: Cat# BQE-M0111) following the manufacturer’s instructions. All samples and standards were run in duplicate.

### 4.5. RNA Extraction and qPCR

Total RNA was extracted from lung tissues using TRIzol reagent (ABclonal, Wuhan, Hubei, China, Cat# RK30129). Briefly, lung tissue samples were homogenized in TRIzol reagent, phase separation was performed by adding a chloroform substitute (Beyotime, Shanghai, China, Cat# R0006), and RNA was precipitated with isopropanol (Macklin, Shanghai, China, Cat# I811932), washed with 75% ethanol (Macklin, Shanghai, China, Cat# E809059), and dissolved in RNase-free water. First-strand cDNA was synthesized from 1 μg of total RNA using ABScript III Reverse Transcriptase (ABclonal, Wuhan, Hubei, China, Cat# RK20408) according to the manufacturer’s instructions. Quantitative real-time PCR (qPCR) was performed using Genious 2X SYBR Green Fast qPCR Mix (ABclonal, Wuhan, Hubei, China, Cat# RM21204) on an Archimed X6 system (RocGene, Beijing, China) according to the manufacturer’s instructions. The thermal cycling conditions were as follows: initial denaturation at 95 °C for 30 s, followed by 40 cycles of 95 °C for 5 s and 60 °C for 30 s, and a final melt curve analysis. All reactions were performed in triplicate. GAPDH was used as an internal control. Relative mRNA expression levels of target genes were normalized to GAPDH and calculated using the 2^−ΔΔCt^ method [64,65], in which ΔCt = Ct(target) − Ct (GAPDH) and ΔΔCt = ΔCt(treated) − ΔCt(control). All primer sequences used in this study are provided in the Supplementary Materials.

### 4.6. Western Blotting

Total protein was lysed in the Western and IP Cell Lysis Buffer (Beyotime Biotechnology, Shanghai, China; Cat. No. P0013) containing 20 mM Tris (pH 7.5), 150 mM NaCl, 1% Triton X-100 and a mixture of protease and phosphatase inhibitors (sodium pyrophosphate, β-glycerophosphate, EDTA, Na_3_VO_4_ and leupeptin). After centrifugation at 12,000× *g* for 10 min at 4 °C, the supernatant was collected, and protein concentrations were determined using the BCA method. Protein samples were mixed with M5 Odorless 5× SDS-PAGE Loading Buffer (Mei5 Biotechnology, Beijing, China; Cat. No. MF1227-10) containing 250 mM Tris-HCl (pH 6.8), 10% SDS, 0.5% bromophenol blue, 50% glycerol and 1% DTT, denatured by heating at 95 °C for 10 min, and then separated by SDS-PAGE and transferred onto PVDF membranes. The membranes were blocked with 5% non-fat milk for 1 h, then incubated with primary antibodies (see Supplementary Materials for details) overnight at 4 °C, followed by incubation with HRP-conjugated secondary antibodies for 1 h at room temperature. Protein bands were visualized using chemiluminescence, captured with a gel imaging system, and quantified using ImageJ software.

### 4.7. Thermal Proteome Profiling

A549 cells at 90–100% confluence were harvested and lysed in Western/IP lysis buffer containing protease and phosphatase inhibitors. The extracted protein lysates were then allocated into control and treatment groups. The control group received DMSO, whereas the treatment group was exposed to graded concentrations of VK3. All samples were incubated at room temperature for 1 h, followed by heating at 52 °C for 4 min. After cooling to ambient temperature, soluble proteins were obtained by centrifugation at 20,000× *g* for 20 min at 4 °C and subsequently analyzed by Western blotting. Proteomic profiling was carried out by Shanghai OE Biotech Co., Ltd. (Shanghai, China).

### 4.8. Protein Expression and Purification

The coding sequences of human *TANK* and *TBK1* were individually cloned into the pET-28a (+) vector using NdeI and XhoI restriction sites. Each recombinant plasmid was transformed into *E. coli* BL21(DE3) competent cells. Positive clones were selected, cultivated in LB medium at 37 °C to an OD600 of 0.6–0.8, and induced with 0.5 mM IPTG at 25 °C overnight. Cells were harvested by centrifugation, resuspended in lysis buffer, and disrupted by ultrasonication. The lysate was cleared at 3500 rpm for 20 min at 4 °C, and the supernatant was loaded onto pre-equilibrated Ni-NTA resin (Beyotime) to obtain non-denatured proteins. Elution was performed with 200 mM imidazole. The eluate was then ultrafiltered to replace the imidazole solution with PBS. Protein purity was analyzed by SDS-PAGE, and concentrations were determined using a BCA protein assay kit (Beyotime, Shanghai, China, Cat# P0012S).

### 4.9. Direct Binding Assays

The direct binding interactions between VK3/VK2 and TANK/TBK1 were assessed using differential scanning fluorimetry (DSF), cellular thermal shift assay (CETSA), and biolayer interferometry (BLI). All assays were carried out according to our previously established protocols [66]. In brief, DSF monitored thermal stability shifts in the purified proteins, CETSA evaluated target engagement in A549 cell lysates, and BLI determined the equilibrium dissociation constants (*K*_d_) for the protein–compound interactions.

### 4.10. Molecular Docking and Molecular Dynamics Simulations

The crystal structure of human TBK1 (PDB ID: 4IM2, resolution 2.50 Å) was retrieved from the RCSB Protein Data Bank, and the ATP-binding pocket within the kinase domain was defined as the docking site [67]. The three-dimensional structure of VK3 (PubChem CID 4055) was retrieved from PubChem. Molecular docking was performed using AutoDock (v1.5.7), and the protein and ligand were prepared by removing water molecules and the co-crystallized ligand, adding polar hydrogens, assigning Gasteiger charges, and defining rotatable bonds (PDBQT format). To validate the docking protocol, the co-crystallized inhibitor BX7 was re-docked into the same binding pocket, and the docked pose superimposed well with the native conformation (RMSD < 2.0 Å), confirming the reliability of the grid box and docking parameters. The top-ranked pose was visualized in PyMOL (v2.5.2) to assess hydrogen bonds and hydrophobic interactions.

Molecular dynamics simulations were carried out using the Amber 24 package. The ff19SB force field [68] was used to parameterize the system, and the complex was solvated using the TIP3P water model, with Na^+^ and Cl^−^ counterions added at a final concentration of 0.15 M by the Monte Carlo method to neutralize the system. After energy minimization, the system was heated from 0 K to 310.15 K (37 °C) over 500 ps in the canonical (NVT) ensemble and pre-equilibrated at 310.15 K (37 °C). A 200 ns production run was subsequently performed in the isothermal–isobaric (NPT) ensemble at 1 bar with periodic boundary conditions maintained throughout, using a 2 fs time step. All covalent bonds involving hydrogen atoms were constrained by the SHAKE algorithm. Trajectories were analyzed using AmberTools 23 for root-mean-square deviation (RMSD), root-mean-square fluctuation (RMSF), and hydrogen bond occupancy.

### 4.11. Single-Cell Data Acquisition and Drug2cell Analysis

Single-cell transcriptomic data from lung tissue were obtained from the GEO database (accession number GSE128033) [13]. The manually annotated dataset contained 55,015 cells from pulmonary fibrosis samples, covering ten major cell types (epithelial cells, fibroblasts, macrophages, monocytes, mast cells, endothelial cells, smooth muscle cells, B cells, T cells, and natural killer cells). After quality control filtering based on mitochondrial and ribosomal gene content, 2603 high-quality genes were retained. Gene expression values were normalized and batch effects were corrected using standard procedures. Drug activity scores for 2381 compounds were calculated using the Drug2cell method [11,12], which quantifies per-cell enrichment of drug target gene sets through a gene set enrichment analysis (GSEA) framework. Briefly, known target genes of menadione were retrieved from the latest ChEMBL database release. For each cell, Blitz GSEA was performed on the target gene set to generate a normalized enrichment score (NES), which was then transformed into a drug sensitivity score. To compare sensitivity scores across cell types, two-sided Wilcoxon rank-sum tests were applied, and the false discovery rate (FDR) was controlled using the Benjamini–Hochberg procedure, with statistical significance defined as FDR < 0.05.

### 4.12. scRNA-Seq Data Generation and Processing

A microwell-based single-cell RNA sequencing platform (10x Genomics) was used for transcriptomic analysis of mouse lung tissue samples. Single-cell suspensions were loaded onto microwell chips and combined with oligonucleotide beads carrying cell barcodes and unique molecular identifiers (UMIs). Cells were lysed and mRNA was captured. cDNA was synthesized by reverse transcription. Libraries were constructed and sequenced on an Illumina platform with paired-end 150 bp reads. Raw data were aligned to the mouse reference genome (mm10) using Cell Ranger v7.2 to generate expression matrices. Quality control was performed with Seurat v5.0.0. Low-quality cells (gene number < 500, mitochondrial gene ratio > 10%) and low-expression genes were filtered out. Data were normalized, log-transformed, and scaled. Highly variable genes were selected for principal component analysis (PCA). Batch effects were removed using Harmony. Dimensionality reduction and clustering were performed with UMAP. Cell types were annotated based on marker genes for each cluster (Wilcoxon test, FDR < 0.05) and comparison with known cell marker databases [69].

### 4.13. Statistical Analysis

Normality of the data was assessed using the Shapiro–Wilk test, and homogeneity of variances was evaluated by Levene’s test. Datasets that satisfied the normality assumption (*p* > 0.05) were analyzed using parametric tests: one-way analysis of variance (ANOVA) was employed for comparisons among three or more groups, with Tukey’s test used for multiple comparisons when variances across groups were equal and Dunnett’s T3 test applied when variances were unequal; comparisons between two groups were performed using a two-tailed unpaired Student’s *t* test. Datasets that did not satisfy the normality assumption were analyzed using non-parametric tests: the Kruskal–Wallis test followed by Dunn’s post hoc test was used for comparisons among three or more groups, and the Mann–Whitney U test was used for comparisons between two groups. All experiments included at least three independent biological replicates. Data are presented as mean ± standard deviation (SD). Statistical significance was set at *p* < 0.05.

## 5. Conclusions

In summary, this study was designed to characterize the direct interaction between VK3 and TBK1 and explore whether VK3 could augment the antifibrotic effects of PFD. Our results identify VK3 as a direct TBK1-targeting agent that enhances the antifibrotic efficacy of pirfenidone both in vitro and in vivo. Additional experiments using VK3 alone further demonstrate that its antifibrotic activity is independent of metabolic conversion to vitamin K2. Orthogonal target validation assays together with functional TBK1 knockdown experiments collectively validate TBK1 as a promising therapeutic target for antifibrotic drug discovery. Consistent with our initial hypothesis, these findings establish a target-validated foundation for lead optimization of naphthoquinone scaffolds, with the goal of developing safer and more potent agents against IPF. This work also supports further preclinical evaluation of VK3 as an adjunct agent for PFD-based combination therapy. Moving forward, planned follow-up studies include validating these findings in human primary alveolar cells and stem cell-derived alveolar organoids, delineating the precise mechanism by which TBK1 modulates STAT3 phosphorylation, characterizing VK3 pharmacokinetics and long-term safety, and performing structure-guided optimization of the naphthoquinone scaffold to generate improved TBK1 inhibitors with higher affinity and lower cytotoxicity.

## Data Availability

Proteomics data generated in this study have been deposited in the ScienceDB database and are publicly available under DOI: 10.57760/sciencedb.46073 [70]. Single-cell RNA sequencing data have been deposited in the CNCB-NGDC database under accession number PRJCA071531 [71]. All other data supporting the findings of this work are available from the corresponding author upon reasonable request.

## Supplementary Materials

The following supporting information can be downloaded at: https://www.mdpi.com/article/10.3390/ijms27219739/s1.
