# Supplementary material for: Vitamin K3 (Menadione) Targets TBK1 to Enhance the Efficacy of Pirfenidone in a Mouse Model of Bleomycin-Induced Pulmonary Fibrosis
Source: Int J Mol Sci. 2026 Oct 30;27(21):9739. doi: 10.3390/ijms27219739 (PMC13762912; doi:10.3390/ijms27219739)
Supplement: Supplementary file 1 [file ijms-27-09739-s001.zip › ijms-4592944-supplementary.pdf]

Supplementary Material

Vitamin K3 (Menadione) Targets TBK1 to Enhance the Efficacy of Pirfenidone in a Mouse Model of Bleomycin-induced Pulmonary Fibrosis

Yanan Wang [1], Junxian Cao [2], Yingxin Zhou [3], Xuxia Cao [2], Shiwen Deng [2], Yu Li [1], Caifeng Li [2], Shihuan Tang [1, *] and Peng Chen [2, *]

1 State Key Laboratory for Quality Ensurance and Sustainable Use of Dao-Di Herbs, Institute of Chinese Materia Medica, China Academy of Chinese Medical Sciences, Beijing 100700, China;

2 Experimental Research Center, China Academy of Chinese Medical Sciences, Beijing 100700, China;

3 Jiangxi Province Key Laboratory of Traditional Chinese Medicine Pharmacology, Institute of Traditional Chinese Medicine Health Industry, China Academy of Chinese Medical Sciences, Nanchang 330115, China;

* Corresponding author:

1. Shihuan Tang (tshuan800@126.com), Institute of Chinese Materia Medica, China Academy of Chinese Medical Sciences, Beijing 100700, China

2. Peng Chen (chenpeng@merc.ac.cn), Experimental Research Center, China Academy of Chinese Medical Sciences, Beijing 100700, China.

SUPPLEMENTARY METHODS

Cell Counting Kit-8 Assay

Cell viability was assessed using the CCK-8 reagent. Cells were seeded in 96-well plates at an appropriate density and allowed to adhere overnight. The next day, cells were treated with various concentrations of the test compound or vehicle for 48 h. After treatment, 10 μL of CCK-8 solution was added to each well and incubated for an additional 2 h at 37 °C. The absorbance at 450 nm was measured using a microplate reader (BioTek Synergy2). Cell viability was calculated as the percentage relative to the vehicle-treated control group after subtracting background absorbance.

Immunofluorescence (IF) Assay

Cells were seeded on glass coverslips (Biosharp) and treated with the drugs for a defined period. After treatment, cells were washed twice with PBS, fixed with 4 % paraformaldehyde (Beyotime) for 15 min at room temperature, and then permeabilized with 0.1 % Triton X-100 (Beyotime) for 10 min. Non-specific binding was blocked by incubating with 5 % bovine serum albumin (BSA, ABclonal) in PBS for 1 h at room temperature. Subsequently, cells were incubated with primary antibodies diluted in blocking buffer overnight at 4 °C. After washing three times with PBS, cells were incubated with Alexa Fluor-conjugated secondary antibodies for 1 h at room temperature in the dark. Nuclei were counterstained with DAPI for 5 min. Coverslips were mounted onto slides with an anti-fade mounting medium and images were acquired using a fluorescence microscope.

Cellular Thermal Shift Assay (CETSA)

A549 cells were harvested and lysed. After centrifugation, the supernatant containing soluble proteins was collected. The cell lysate was then divided into aliquots and heated at different temperatures (ranging from 37 °C to 72 °C) for 4 min in a thermal cycler. Following heating, the samples were cooled to room temperature for 3 min and centrifuged at 20,000×g for 5 min at 4 °C to separate soluble proteins from precipitated aggregates. The supernatants were collected and analyzed by Western blotting using the indicated antibodies.

Lentiviral shRNA-Mediated Knockdown of TBK1

Lentiviral vectors containing short hairpin RNA (shRNA) targeting TBK1 were constructed for gene knockdown. shRNA sequences targeting TBK1 were designed and cloned into the pLKO.1-puro lentiviral vector (General Biosystems (Anhui) Corp. Ltd.). Lentiviral particles were produced by co-transfecting HEK293T cells with the shRNA-containing transfer vector, together with packaging plasmids and envelope plasmid, using a lentiviral packaging kit (Beijing Dona Pharmaceutical Technology Co., Ltd.). Virus-containing supernatants were collected at 48 h and 72 h post-transfection, concentrated by ultracentrifugation, and stored at –80 °C. For transduction, A549 cells were incubated with the concentrated lentivirus for 6 h. After 48 h, stable knockdown cells were selected with puromycin (1 µg/mL) for 5 days. Knockdown efficiency was confirmed by Western blotting or qRT-PCR using specific antibodies or primers against TBK1.

SUPPLEMENTARY FIGURES

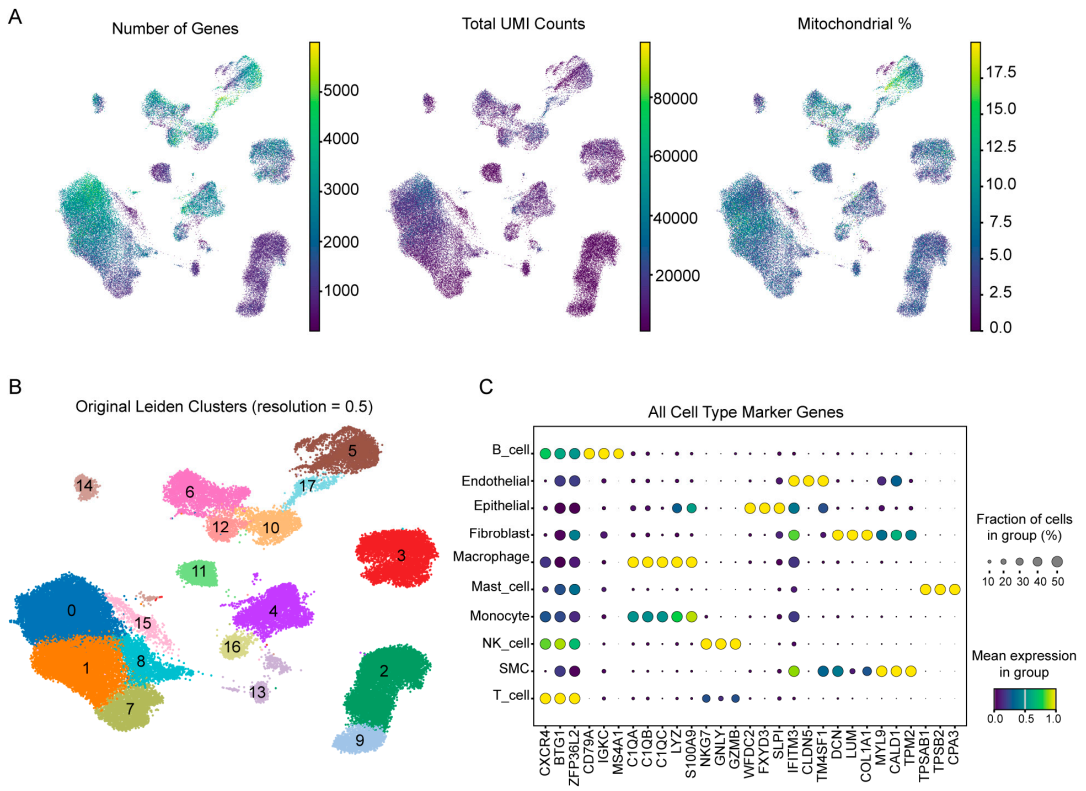

Figure S1 Screening of VK3 by integrating public single-cell sequencing data with the Drug2cell method. A Cells are projected onto a two-dimensional UMAP space and colored by the number of detected genes, total UMI counts, and mitochondrial percentage. **B** UMAP plot of single-cell clusters identified by Leiden algorithm. **C** Dot plot showing expression of marker genes across all major cell types.

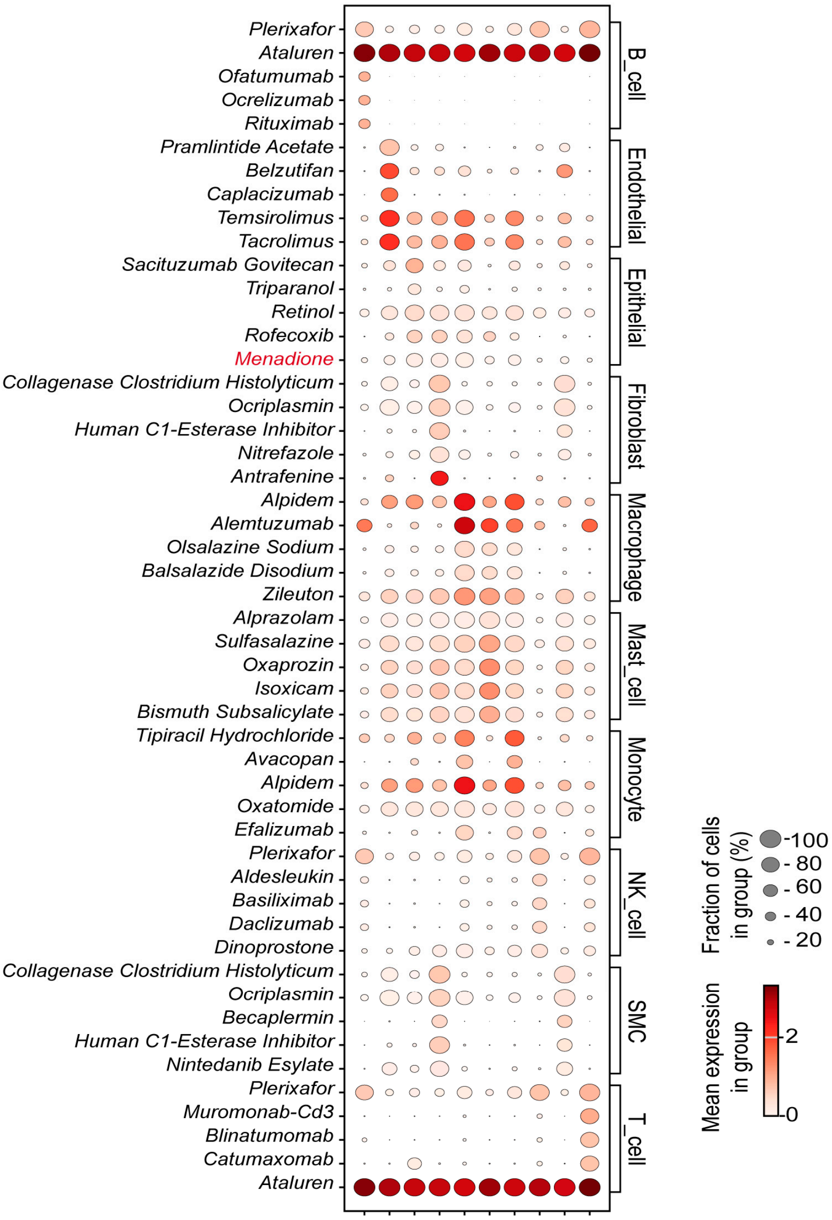

Figure S2. Screening of VK3 based on public single-cell transcriptome data and the Drug2cell algorithm. Bubble plot showing predicted drug sensitivity scores across all ten cell clusters.

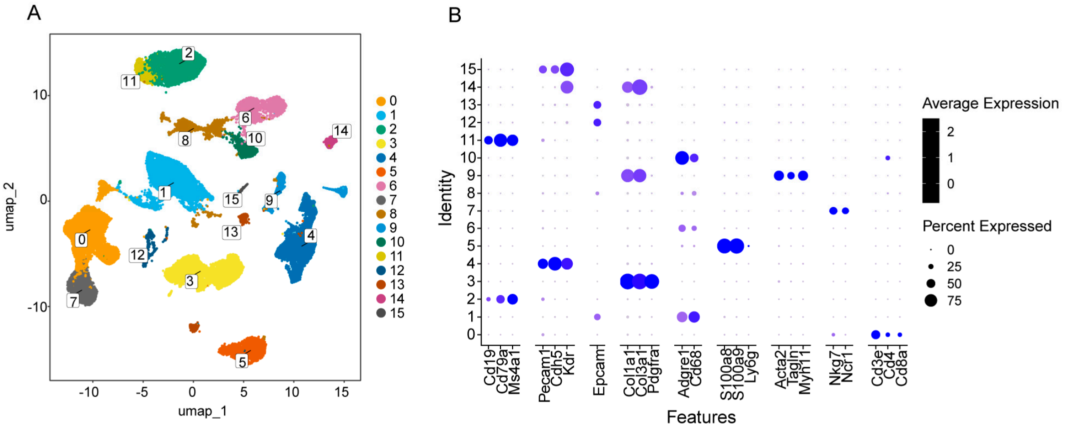

Figure S3 VK3 and PFD remodel the scRNA-seq landscape in fibrotic mouse lungs.

A UMAP visualization of single-cell clusters identified by Seurat**. B** Dot plot showing the expression levels of representative marker genes for the major cell subtype.

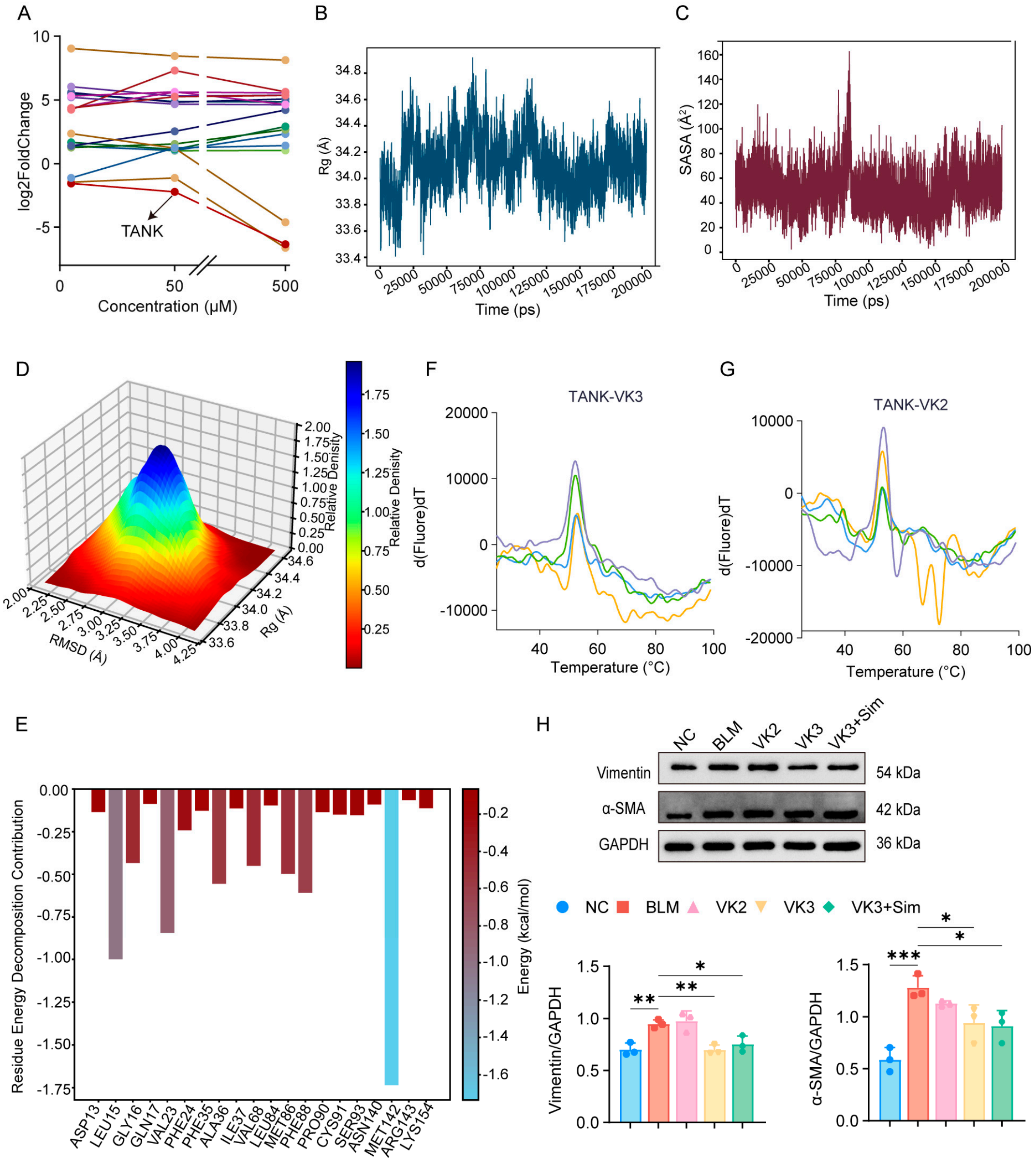

Figure S4 VK3 directly targets TBK1 with high affinity. A TPP results identifying TANK as a potential VK3-interacting protein. **B** Rg of the complex plotted over the entire simulation time. **C** SASA analysis of VK3 bound to TBK1. **D** Three-dimensional free energy landscape of the VK3-TBK1 complex**. E** Per-residue energy contribution analysis of the VK3–TBK1 interaction. **F-G** BLI binding curves of TANK-VK3 (**F**), TANK-VK2 (**G**). **H** Representative Western blots and combined densitometric

quantification of α-SMA and VIM in A549 cells under the indicated treatments. GAPDH was used as the loading control, and band intensities were normalized to GAPDH. *$P < 0.05$, ** $P < 0.01$, *** $P < 0.001$ vs. BLM group.

SUPPELMENTARY TABLES

Table S1 Real-time PCR Primers

Gene name	Forward primer	Reverse primer
GAPDH-H	ACCTTCCCCATGGTGTCTGA	GCTCCTCCTGTTCGACAGTCA
TGFB-H	CCCCTACATTTGGAGCCTGG	GCACGATCATGTTGGACAGC
COL1A1-H	GATTCCCTGGACCTACTAAAGGTGC	AGCCTCTCCATCTTTTTGCCAGCA
COL3A1-H	CGTGGCTCTAATGGCATCAAAGG	ATGTGGTCCAACTGGTCCTCTG
α-SMA-H	CAAAGTCGTGGACAACCAGGCT	GTCCAAGGCTTTGTGTGAGCA
VIM-H	AGGCAAAGCAGGAGTCCACTGA	ATCTGGCGTTCCAGGGACTCAT
FN-H	ACAACACCGAGGTGACTGAGAC	GGACACAACGATGCTTCCTGAG
Snail-H	TGCCCTCAAGATGCACATCCGA	GGGACAGGAGAAGGGCTTCTC
Slug-H	ATCTGCGGCAAGGCGTTTTCCA	GAGCCCTCAGATTTGACCTGTC
Twist-H	GCCAGGTACATCGACTTCCTCT	TCCATCCTCCAGACCGAGAAGG
TBK1-H	CAACCTGGAAGCGGCAGAGTTA	ACCTGGAGATAATCTGCTGTCGA
GAPDH-M	GGCAAATTCAACGGCACAGTCAAG	TCGCTCCTGGAAGATGGTGATGG
TGFB-M	TGATACGCCTGAGTGGCTGTCT	CACAAGAGCAGTGAGCGCTGAA
COL1A1-M	AGGCGAACAAGGTGACAGAGG	GGAGAACCAGGAGAACCAGGAG
α-SMA-M	TCAGGGAGTAATGGTTGGAATGGG	GGTGATGATGCCGTGTTCTATCG

VIM-M	CCGCAGCCTCTATTCCTCATCC	AGTCCACCGAGTCTTGAAGCAG
FN-M	CTTGGTGGCTGTGAAAGGGAAC	CTCGGTGTTGTAAGGTGGAATGG

Table S2 List of antibodies used.

Antibody	WB	IF	Company	Cat.
VIM	1:10000	1:400	Abclonal	A19607
α-SMA	1:1000	1:400	Beyotime	AG8004
FN	1:1000	1:400	Beyotime	AF6912
COL1A1	1:1000	1:400	CST	72026T
TGFB	1:1000	1:400	Selleck	F1016
E-Cadherin	1:1000	1:400	Abclonal	A20798
TBK1	1:1000	1:400	Abcam	ab40676
P-TBK1 (Ser172)	1:1000		Beyotime	AF5959
IRF3	1:1000		Stater	S0B0598
P-IRF3 (Ser396)	1:1000		Beyotime	AF5848
JAK1	1:1000		Stater	S0B1336
P-JAK1 (Tyr1022/1023)	1:1000		Beyotime	AF5857
STAT3	1:1000		Abclonal	A11216
P-STAT3 (Tyr 705)	1:1000		Abclonal	AP1468
GAPDH	1:50000		ABclonal	A19056
β-Actin	1:80000		ABclonal	AC026

Table S3 shRNA targeting sequences for TBK1 knockdown.

shRNA	Sequence
Sh-TBK1-1	TTGTCCATGTGGGAGTTTATA
Sh-TBK1-2	TGATATCAAGCCAGGAAATAT
Sh-TBK1-3	GAAGGGCCTCGTAGGAATAAA
